# Triggering Risk Factors of the Burnout Syndrome in Ob/Gyn Physicians from a Reference Public University of Brazil

**DOI:** 10.5402/2012/593876

**Published:** 2012-12-06

**Authors:** Fátima Ferreira Bortoletti, Ana Maria Teresa Benevides-Pereira, Esdras Guerreiro Vasconcellos, José Oliveira Siqueira, Edward Araujo Júnior, Luciano Marcondes Machado Nardozza, Ricardo Werner Sebastiani, Antonio Fernandes Moron

**Affiliations:** ^1^Department of Obstetrics, Federal University of São Paulo (UNIFESP), 05303-000 São Paulo, SP, Brazil; ^2^Department of Psychology, State University of Maringá (UEM), 87020-900 Maringá, PR, Brazil; ^3^Department of Labor and Social Psychology, Institute of Psychology, São Paulo University (IPUSP), 05508-030 São Paulo, SP, Brazil; ^4^Department of Experimental Psychology, Institute of Psychology, São Paulo University (IPUSP), 05508-030 São Paulo, SP, Brazil; ^5^Centre for Studies and Research in Psychology and Health (Nemeton), 04533-012 São Paulo, SP, Brazil

## Abstract

*Objective*. To identify the risk factors to the development of Burnout Syndrome in Ob/Gyn Brazilian physicians in four dimensions: emotional exhaustion (EE), professional repression (PR), dehumanization (De), and emotional distancing (EmD). *Methods*. A prospective cross-sectional study was realized with 48 Ob/Gyn physicians (12 lecturers, 12 attending physicians, 12 medical residents, and 12 graduate students) from Department of Obstetrics, São Paulo Federal University (UNIFESP). We used a sociodemographic questionnaire focusing on the activities (administrative, educational, healthcare, and research). We applied a Burnout Syndrome Inventory (BSI) composed of two parts: triggering factors (ISB1) and the Burnout Syndrome (ISB2). The ISB1 is composed of two scales: positive organizational conditions (POC) and negative organizational conditions (NOC). The ISB2 is composed of four scales: EE, PR, De, and EmD. *Results*. We observed a rate below and above average to POC and NOC, respectively. The dimensions recorded a level above average to EE, an index at the upper limit of the average to De, a median index to EmD, and a median index to PR. *Conclusions*. The Ob/Gyn physicians are in an area of vulnerability for the development of Burnout Syndrome due to the high level of EE and De, associated with a median index of PR. The high rate of NOC contributes to the triggering of this scenery.

## 1. Introduction

Burnout Syndrome was defined for the first time by Herbert Freudenberger as a condition of fatigue or frustration produced by dedication to a cause expected to give positive results that conflicts with a reality that does not correspond to the professional's expectations [1]. According to Moreno-Jiménez et al. [2], based on this definition, interest became focused on the description of the consequent symptomatology, widely defined in physical, behavioral, emotional, cognitive, and motivational terms. Symptoms would be the result of a poorly adaptive and realistic effort that causes exhaustion due to negligence of one's own needs in favor of full dedication to work. This clinical approach privileges the importance of individual variables in the onset of the syndrome. From a psychosocial perspective, professional wear and tear is a response to chronic stress at work, and results from a process that suffered interference both from individual characteristics and from those of the work environment.

Vasconcellos [3] considers as a main characteristic of healthy and necessary stress (Eustress), the fact that after the excitement phase the body get back to baseline levels. When the body cannot return to its normal state it adapts to high levels, down to Local Adaptation Syndrome (SAL), the physical symptoms disappear and they are replaced by psychosocial symptoms: irritability, social withdrawal, chronic fatigue, impatience, loss of sensation, inability to dialogue, lack of sexual appetite and, in men, impotence, but above all, loss of enjoyment of life, except to work. Despite the fatigue and lack of motivation at this early stage of SAL, we still cannot talk about Burnout Syndrome. The chronicity of this state raises the Burnout Syndrome.

Fernandéz-Lopez et al. [4] recorded that the Burnout Syndrome manifests most frequently in certain professions, and among healthcare professionals there is an incidence of 20 to 50%, second only to police officers and teachers. If we consider healthcare professionals who also develop daily teaching activities, these are second only to police officers. The symptoms are characterized by a drop in productivity, increased absenteeism, increased turnover, as well as work-related accidents, decreased quality of work, besides nurturing a negative image of the work place. According to Gabbe et al. [5], physicians in the gynecology and obstetrics specialty have an increased incidence of burnout syndrome due to their close relationship with their clients. Other common factors in the gynecologist/obstetrician routine collaborate enormously, such as the lack of human resources and materials, inadequate physical and environmental conditions, staggered shifts, and the frequent absence of organizational support [6].

Therefore, the objective of this study is to analyze the factors of the Burnout Syndrome in a sample of Brazilian gynecologists/obstetricians who work in obstetric care, focused on emotional exhaustion, dehumanization, emotional distancing, and professional repression. 

## 2. Methods

A prospective cross-sectional study was carried out with 48 Brazilian doctors, from August 2010 to February 2011. This study was approved by the Research Ethics Committee of the Federal University of São Paulo (UNIFESP), and the participants who agreed to voluntarily participate signed the informed consent form. The sample of 48 gynecologists/obstetricians was divided into 12 lecturers, 12 attending physicians, 12 medical residents, and 12 graduate students; all professionals in the Department of Obstetrics of the UNIFESP. 

A sociodemographic form was applied requesting that the participants provide information about the area in which they worked, whether administrative, education, direct patient care, or research. Furthermore, the Burnout Syndrome Inventory (BSI), validated in Brazil by Benevides-Pereira [7] was applied. This questionnaire is composed of two parts: triggering factors (ISB1) and the Burnout Syndrome (ISB2). The triggering factors are organizational factors that predispose towards the onset of the syndrome, that is, variables of vulnerability to stress and to Burnout Syndrome. This part comprises two scales: positive organizational conditions (POC) and negative organizational conditions (NOC), each one with eight items. Each item presents with five response alternatives: 0: never, 1: rarely, 2: sometimes, 3: frequently, and 4: very frequently. The Burnout Syndrome (ISB2) module evaluates the syndrome per se, and is composed of four scales: emotional exhaustion (EE) (5 items) professional repression (PR) (6 items) dehumanization (De) (5 items), and emotional distancing (EmD). Each item has five response alternatives: 0: never,1: a few times a year, 2: a few times a month, 3: a few times a week, and 4: every day. Each interviewee, anonymously and in a private room, took up to 20 minutes to complete the form and the BSI questionnaire. After concluding the answers, the envelopes were sealed and the principal investigator (FFB) had no access to the content of the responses until the end of data collection. 

The data were transferred to a spreadsheet of the Excel 2007 program (Microsoft Corp., Redmond, WA, USA) and analyzed by the SPSS program (SPSS Int., Chicago, IL, USA) of Windows version 17.0. Descriptive analyses were performed (mean, median, standard deviation, and maximum and minimum values), mean comparison analysis (ANOVA and Student's *t* test), and correlation analysis (Pearson correlation coefficient—*r*). 

## 3. Results

The sample proved to be balanced in terms of sex, with 50% females and 50% males, age varying between 25 and 61 years. As to the marital status, 31.3% of the participants were single, 64.6% were married or maintained a stable union, and 4.2% were separated, widowed, or divorced. Regarding children, 54.2% of the participants had no children, 12.5% had one, 25.0% had two, 6.3% had three, and 2.1% had five children. As to time since graduation, we found a variation between one and 36 years, with the highest percentage (10.6%) found between 3 and 4 years, followed by (8.5%) between 24 and 28 years. As to activities performed, all participants were engaged in patient care, 50% in a position of teaching, 20.8% performed administrative functions, and 47.9% worked in their subspecialty of maternal-fetal medicine. All participants worked at a public hospital, and 60.4% also worked at private hospitals; 4.2% developed activities in healthcare insurance plans, 60.4% worked in private medical offices, and 41.7% worked with ultrasound in Obstetrics. As to work load, the shift loads varied from 12 to 54 hours a week; in that, 27.1% worked 24 hours a week and 43.8% did not do hospital shifts. As to daily work load, 46.7% of the participants recorded a mean of 12 hours and 28.9% pointed to a mean of 10 hours.

The evaluation of ISB1 questionnaire indicated data related to the POC of the institution, recording that frequently/very frequently 41.7% of the responders had support from their superiors; 58.3% were a part of a team; 75.0% received collaboration from colleagues; 64.6% felt respected in their work; 43.8% perceived transparency in rules; 31.3% felt flexibility in performing their work; 58.3% considered their work environment as pleasant and safe.

As to the NOC results, we recorded that frequently/very frequently 58.3% of the responders considered they waste a lot of time with bureaucracy; 14.6% did not trust their colleagues; 29.2% had no time to reflect on their work; 54.2% believed that friends of their bosses were privileged; 22.9% considered that competence is less important than submission; 20.8% feared being the “next victim”; 37.5% perceived the tense environment; 14.6% noticed a climate of intimidation in their work environment.

As to the correlation among the four groups (lecturers, attending physicians, medical residents, and graduate students) and the POC and NOC, we observed a negative correlation in the lecturer and attending physician groups, with no statistical significance (*r* = −0.531 and *P* = 0.075 and *r* = −0.224 and *P* = 0.484, resp.). However, for the resident and graduate student groups, we noted a negative correlation with statistical significance in POC and NOC (*r* = −0.727 and *P* = 0.007 and *r* = −0.683 and *P* = 0.014, resp.). 

For the EE dimension, we recorded that a few times a week/every day 47.9% participants felt with no energy after a day of work; 52.1% woke up feeling tired; 14.6% had no stamina for anything else; 27.1% needed to put forth great effort to get up and go to work; 29.2% felt that work consumes all their energy. For the EmD dimension, we recorded that a few times a week/every day 41.7% of the participants had no patience with some of the people at work; 25.0% perceived that they had distanced themselves emotionally from people, maintaining impersonal contacts at work; 27.1% avoided personal contact at work, confirmed by the 31.3% who avoided close contact. For the De dimension, we recorded that a few times a week/every day 33.3% of the participants considered they had become “tougher” after some time on the job; 14.6% became insensitive to the problems of people at work; 25.0% felt they had to toughen-up to keep this job; 25.0% became more “technical” and less “human” in their work. For the PR dimension, we recorded that a few times a week/every day 75.5% conducted the activity they had always desired; 77.1% felt that this work is appropriate for them, 85.4% identified with their work; 79.2% perceived their work as important.

In the lecturer group, we found a positive correlation (*r* = 0.721) with a level of significance (*P*) 0.008 between the dimensions of De and EmD. The attending physician group showed a positive correlation (*r* = 0.813) which is significant (*P* = 0.001) between the dimensions of De and EmD. In the group of medical residents, we found the highest positive correlation of the sample (*r* = 0.931), with a significance level lower than 0.001 between dimensions EE and De and a positive correlation (*r* = 0.581) with a significance level of 0.05 between dimensions De and EmD. In the graduate student group, we found three positive and significant correlations, in which the first was between dimensions of EE and De (*r* = 0.899) with a significance level *P* = 0.000; the second was between EE and EmD (*r* = 0.665) with a significance level *P* = 0.018; the third between dimensions De and EmD (*r* = 0.802) with a significance level *P* = 0.002.

Among the positive aspects reported by the groups within their professional area of work, the lecturer group mentioned the ample practice of Medicine (45%) and participating in the preparation of new physicians (65%). For the attending physician group, aspects mentioned were the contact with students and residents, and consequently, the possibility of teaching (20%), and the possibility of updating (70%). For the resident group, the possibility of helping people (33.3%). For the graduate student group, the possibility of collaborating in technical and cultural growth and relationships with patients were reported by 45%. As to negative aspects, 25% of the lecturer group mentioned the occurrence of intrigues among members of the faculty of the department. For the attending physician group, 25% reported a lack of material and precariousness of comfort conditions for the physician and the Emergency Unit as to physical space and hygiene. For the resident group, 25% reported interference of nursing professionals, followed by 20% who reported lack of resources and very poor work site conditions. For the graduate student group, 35% reported lack of planning and organization.

Table 1 shows the mean of scores obtained by ISB1 in POC and NOC with the groups of lecturers, attending physicians, residents, and graduate students. For each one of these four groups, the mean POC score was lower than average, while for NOC the mean score was above average, considering the reference standard proposed by Benevides-Pereira [7].  Table 2 shows the mean of ISB2 scores for the four dimensions, EE, De, EmD, and PR, with the groups of lecturers, attending physicians, residents, and graduate students. For the lecturer group, we noted a median score for EE, at the upper limit for De, above average for EmD, and median for PR. For the group of attending physicians, we found an above average score for EE, median for De, above average for EmD, and median for PR. For the medical resident group, we observed an above average score for EE, above average for De, above average for EmD, and median for PR. For the graduate student group, we noted an above average score for EE, at the upper limit for De, above average for EmD, and median for PR, considering the reference standard proposed by Benevides-Pereira [7].

## 4. Discussion

In the Department of Obstetrics, (UNIFESP) we recognize the exercise of leadership from the medical resident of second year (R2) which is higher than medical resident of first year (R1), the medical resident of third year (R3) than the R2 and so on through the medical resident of fourth year (R4), Attending physician, Professor Head of Discipline, Professor Head of Department, and Full Professor. The choice of four groups was intentional so we could not only compare differences among them, but also have an overview of the physicians who work at our Department. According to literature, the accumulation of activities, time since graduation, and hospital shifts are factors that interfere in the work routine, and that is why we were interested in dividing the participants by category, since activities differ in some variables. 

Analyzing POC among the four groups, no significant differences were found, which suggests that the sample group has a homogeneous perception of the benefits that the Department offers. We know that belonging to a group and being able to count on that group is an important support factor for individuals. However, we were concerned with the fact that only 31% mentioned flexibility for developing their work, and we see in this a subjacent negative factor. This issue becomes more significant when considering that only 43% sense transparency regarding rules and support from their heads, which would undermine the positive conditions that do exist. This leads to the possibility of a work environment in which, despite all individuals perceiving what goes on, not all enjoy the same conditions. 

Analyzing NOC among the four groups, again we found no significant differences, which leads to believe that the sample group perceives in an equally homogeneous manner the same conditions. Two issues stood out: the excessive time spent with bureaucracy that bothers 58% of the participants, time that could be used for updating/refresher courses or even for resting; 54% who felt that “friends of the boss” are privileged. Despite not being significant, once again, this suggests a division in the work environment which is highly deleterious for the development of functions. If we consider that the entire institution functions as a system and the whole system is a large gear in which each individual represents a piece of this mechanism, the division of the group may be seen as a condition that limits its function as a whole, hindering all involved. 

In the groups of lecturers and attending physicians, there was a negative correlation between POC and NOC, although this correlation was not significant, and therefore, PCO and NOC are independent. Nevertheless, in spite of the fact that this correlation is not statistically significant, various authors agree that organizational conditions interfere directly in the performance and health of the professional, and for this reason, despite not being found in this sample, one can suppose that an increase in POC may trigger elements favorable to the work activity of lecturers and attending physicians. Maslach and Leiter [8] defend the idea that the solutions should be based on the social context of the work site, a position also defended by Bleger [9], who points out the importance of using the resources available.

A different view is found in the groups of medical residents and graduate students in which a significant negative correlation exists between POC and NOC. In face of this, we can infer that as one increases, the other diminishes, showing that these levels are unstable and that alterations in the dynamics of function within the work environment could have positive or negative reflections on the work climate. In the latter, the consequences may be deleterious and even compromise the permanence of these people in the department. However, if we consider this correlation negative, it signals that investment in POC may translate into a drop in NOC and might lead to increased satisfaction in the activity performed, thus instigating motivation and interest in work.

Nogueira-Martins [10] indicate that the professions most subject to Burnout are those that entail constant interpersonal relationships. Tending to the demands of the external clientele of the department is an arduous task, not only regarding the morbid reality that accompanies it, but also in terms of the precarious institutional conditions these professionals work under; these are attending physicians who toil under unwarranted working conditions and are responsible for the demands of a clientele that frequently needs careful examination. Boyacian and Camano [11] alert to the increased vulnerability of medical activity carried out at a level of urgency and emergency, which expose the professional to intense physical and psychic wear and tear, besides increasing the risk of medical errors and possible lawsuits. In the group of medical residents, it is vital to point out some aspects that may function as aggravating agents when the individuals have an intense work load. This group is responsible for a clientele for which they often do not feel prepared, besides having gone through intense stress in procuring a vacancy in medical residency. We know that frequent alternations of functions require of the professional psychic flexibility in which the emotional and physical wear and tear many times become unbearable [12, 13].

Comparing our results to the means recommended by the BSI [7] as standard reference, we have homogeneity among the four groups, in which the POC are below average and the NOC are above average. Moreno-Jiménez et al. [6] emphasize the importance of work conditions both in preventing and in triggering burnout. In face of this panorama, we suggest a careful and focused examination on the part of healthcare institutions, with the purpose of altering this unfavorable dynamic; this task is not easy, but it is extremely necessary.

Despite the singularities of each group, we can observe that the participants point to the correlation between De and EmD, variables that are highly damaging to the mental health of professionals. Associating these correlations with the means of our participants, as per the reference proposed by Benevides-Pereira [7], we identified a scenario that merits unique attention, since the general average points to an above average score in all these dimensions (EE, De, and EmD).

Considering the four groups, we found four aspects that were cited with greater frequency and that proved to have a positive effect at work: continued learning and updating, feeling useful, the possibility of amply practicing Medicine, and participating in training other physicians. These elements could be explored by managers to stimulate greater development of their professionals. Considering the four groups, we found four aspects that were cited with greater frequency and that proved to have a negative effect at work: interpersonal conflicts, lack of physical and human resources, excessive work load, and lack of organization and planning.

Interpersonal conflicts occurred in 23 participants, almost half of the sample. We considered this datum relevant not only due to its incidence, but especially since it is an aspect deemed a fundamental variable for the onset of burnout. According to Pontes et al. [14], the activities that entail interpersonal relationships are those that expose the professionals to a greater risk for this syndrome. Of the total 23 participants, 11 are a part of the medical resident group, that is, those that suffer the most with these conflicts are the ones who have the least mastery of the profession, which naturally generates discomfort and lack of confidence. 

The lack of physical and human resources was felt in a similar manner among the groups of lecturers, attending physicians, and residents. Gil-Monte and Peiró [15] and Moreno-Jiménez [5] indicate working conditions as an important factor, which under inadequate conditions collaborate in triggering burnout. The complaint as to excessive work load was evident primarily among the medical residents. Gil-Monte and Peiró [15], Maslach et al. [16], and Vega and Urdániz [17] point to the excessive work load as a damaging factor that compromises the quality of work and of physical and psychic health of the professional. We know that after a hospital shift of work, the chances for medical errors increase. In this study we did not directly investigate the consequences that these conditions bring to personal and social lives.

This panorama suggests an important level of exhaustion, which is incompatible with healthy work activity. One must question if these people are not investing too much time in work due to unique undisclosed issues, as they were not the object of study in this paper. However, we must be attentive to the possibility of NOC composing the critical condition of our participants, which is possible considering the scores previously described. According to Leiter [18], these symptoms are precursors of the Burnout picture, in which the individual feels he/she no longer has anything to offer to the job. In sum, we present the results of triggering factors of the Burnout Syndrome in physicians who are gynecologists/obstetricians at our institution. We believe that these results will serve as a reference for the development of prevention mechanisms of this syndrome. Future studies, with larger samples and at other reference centers are necessary to confirm the true triggering factors of the Burnout Syndrome in gynecologists/obstetricians.

## Figures and Tables

**Table 1:** Mean of scores obtained by the Burnout Syndrome Inventory (ISB1) in negative and positive organizational condictions.

	POC		NOC	
	Mean	Standard deviation	Mean	Standard deviation
L	18.92	4.188	17.67	4.997
AP	20.58	5.089	13.58	4.870
GS	21.17	4.988	15.00	6.135
R	19.83	2.758	16.50	3.580
Sample mean	20.13	4.301	15.69	5.062
Study mean*	22–26	—	08–13	—

L: Lecturer, AP: Attending physician, GS: Graduate student, R: Medical resident, POC: Positive organizational condictions, NOC: Negative organizational condictions.

*Student's *t* test.

**Table 2:** Mean of scores obtained by the Burnout Syndrome Inventory (ISB2) in the four dimensions of exhaustion, dehumanization, emotional distancing, and professional repression.

	EE		De		EmD		PR	
	MD	SD	MD	SD	MP	SD	MD	SD
L	7.42	6.515	7.00	3.490	10.17	3.433	12.17	1.193
AP	10.33	3.939	5.67	3.499	8.75	5.083	10.75	3.571
GS	10.25	5.225	6.75	4.309	8.17	5.219	11.83	1.850
R	10.08	2.843	8.00	3.330	7.92	4.461	11.67	2.015
Sample mean	9.52	4.833	6.85	3.655	8.75	4.541	11.60	2.313
Study mean*	04–09	—	04–07	—	02–06	—	10–15	—

EE: Emotional exhaustion, De: Dehumanization, EmD: Emotional distancing, PR: Professional repression, L: Lecturer, AP: Attending physician, GS: Graduate student, R: Medical resident, MD: Mean, SD: Standard Deviation.

*ANOVA test.
